# Transcriptome profiling of a beach-adapted wild legume for dissecting novel mechanisms of salinity tolerance

**DOI:** 10.1038/sdata.2018.290

**Published:** 2018-12-11

**Authors:** Hengyou Zhang, Christine Zuelsdorf, Darin Penneys, Shoujin Fan, Janice Kofsky, Bao-Hua Song

**Affiliations:** 1 Department of Biological Sciences, University of North Carolina at Charlotte, Charlotte, NC 28223, USA; 2 Department of Biology & Marine Biology, University of North Carolina at Willington, Willington, NC 28403, USA; 3 Key Laboratory of Plant Stress Research, College of Life Science, Shandong Normal University, Jinan, Shandong 250014, China

**Keywords:** Plant ecology, Salt, Transcriptomics, Gene expression analysis, RNA sequencing

## Abstract

*Strophostyles helvola* is a close relative to common bean (*Phaseolus vulgaris*) and inhabits both coastal and non-coastal regions in North America. However, the mechanism of saline adaptation in *S. helvola* remains unclear. A transcriptome profiling would facilitate dissecting the underlying molecular mechanisms in salinity-adapted *S. helvola*. In this study, we reported the RNA-seq analyses of two genotypes (a salt-tolerant beach genotype and a salt-sensitive inland genotype) of *S. helvola* stressed with salt. *S. helvola* plants were grown in pots and treated with half lethal-guided dose of NaCl solution for 3 h, 24 h, and 7d. The plants supplied with the same amount of water were used as controls. The whole roots sampled from the three time points were equally pooled as one biological replicate, and three replicates were used for library construction and transcriptome sequencing on Illumina Hiseq 2500. The comparative analyses of root transcriptomes presented here provides a valuable resource for discovery of genes and networks involved in salt tolerance in *S. helvola*.

## Background & Summary

Soil salinity is becoming a critical environmental factor constraining plant growth and crop productivity worldwide because a majority of crops cultivated in irrigated arable land are salt sensitive. In addition, an increase of crop production is in high demand to sustain the growing human population, thus imposing a need of crop cultivation in marginal or coastal land. A sustainable and environmental-friendly alternative strategy is to develop salt-tolerant crops that can thrive in saline soils.

Next-generation sequencing of transcriptomes has been widely used to characterize the global expression patterns in various organisms under diverse conditions to facilitate gene discovery and address major questions associated with plant environmental stress responses^1^. In plants, global transcriptome profiling has been performed to elucidate molecular mechanisms of salt tolerance or response, especially in salinity-adapted genotypes mainly in model systems. Recently, more and more reports on non-model species with limited genomic sequence knowledge have been published. For example, the transcriptome analyses on *Ipomoea imperati*, a wild relative of sweet potato tolerant to high salinity, revealed the ABA signalling pathway and two membrane transporter genes^2^. *Sonneratia alba* represents one of the most salt tolerant mangrove species, and RNA-seq on it has identified salt responsive genes with signatures of natural selection^3^. These transcriptomic studies have allowed us to uncover gene expression mechanisms and novel genes beyond what we currently know from model species.

*Strophostyles helvola* (L.) Elliott, belonging to Fabaceae family, is herbaceous annual vine native to North America. *S. helvola* can colonize in wild places, either moist or dry conditions with preference in sandy soils, thus it is also called wild bean or sand bean. In addition, sand bean is a close relative of common bean (*Phaseolus vulgaris* L.), one of the most important grain legumes worldwide providing protein for human consumption and having roles in fixing atmospheric nitrogen^4^. Like other leguminous crops, cultivated common bean is a glycophyte, sensitive to salt, leading to reduced production if grown under salt conditions^5^. Thus far, molecular mechanisms of common bean’s tolerance to salinity was rarely studied^6,7^, thus the underlying mechanisms remain largely unclear. As a close halophyte relative of common bean, a global investigation of molecular response of salinity-adaptive sand bean can be very helpful with revealing the underlying mechanisms toward the goal to develop salt tolerant common bean.

In this study, we presented the transcriptome analyses of two genotypes of *S. helvola* (halophyte vs glycophyte) stressed with over half-lethal doses of NaCl which was not previously reported. We described the detailed procedure of transcriptome profiling for NaCl-treated and non-treated root tissues of a salt tolerant Beach genotype and a salt sensitive Inland genotype, respectively, during the time-course treatment of 3 h, 24 h, and 7d. In total, 30.5 Gb of transcriptome data (fastq.gz) from twelve root tissues were generated. We also presented in detail the analytic methods of how to obtain the raw sequence, quality control, sequence alignment, and differential expression analyses. We conducted data pre-processing to indicate the high quality of our data as visualized with FastQC and robustness of our results using analyses of multidimensional scales (MDS), dendrogram clustering and expression pattern analyses. The comparative expression profiling of the two *S. helvola* genotypes will provide valuable resources of genomic data for studying salinity tolerance in *S. helvola* and others leguminous relatives.

## Methods

### Overview of experimental design

**Figure 1:**
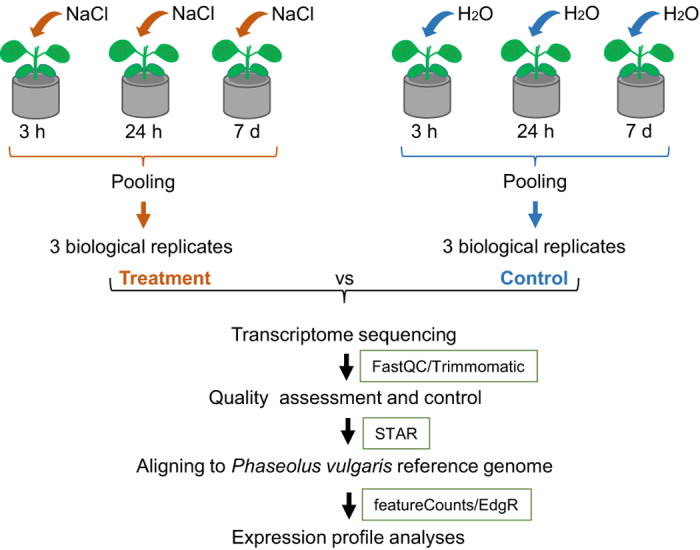
Flowchart of experimental design of this study. A time-course NaCl treatment was used to capture the maximum change in transcriptome of *S. helvola* plants. The transcriptome changes were obtained by a comparison of responses between treated plants with their counterpart controls. Briefly, 10-d old plants were supplied with NaCl solution for treatment, in parallel, the plants supplied with water were used as controls. Three biological replicates per condition were used for transcriptome sequencing. All raw reads were quality controlled prior to aligning to *P. vulgaris* reference genome (*v1.0*). The uniquely aligned reads were used for expression profile analyses.

The whole root tissues that were treated with NaCl and mock (water) were dissected from the plants during the time course investigation at 3 h, 24 h, and 7d. The frozen tissues collected from the three time points were pooled to generate a biological replicate. Total RNA was extracted from the pooled tissues using Qiagen RNeasy mini kit (Qiagen, Valencia, CA, USA) according to the manufacture’s instruction followed by library construction and transcriptome sequencing. The quality-controlled reads were aligned to *P. vulgaris* reference genome, *P. vulgaris_218_v1.0* (https://phytozome.jgi.doe.gov). The resulting *.bam* files were used for differential expression analyses using edgeR^8^. The experiment design and analysis pipeline were shown in Fig. 1.

### Materials and treatment

Two *S. helvola* genotypes were used here for comparative study. One genotype was originally from the sandy soil along the beach in east coast of North Carolina, we named it “Beach genotype” in our study; The other genotype was originally from central Missouri, and we named it “Inland genotype”.

To determine the dose of NaCl that may trigger the tolerance response to salinity, we first tested the median lethal dose (LD50, the salt concentration required to kill 50% of the plants). The beach and inland genotypes were watered every 2 days with increasing increments of 50 mM NaCl solution^9^. We found that two genotypes required different doses of NaCl to cause 50% plants die. For Inland genotype, a final dose 350-mM NaCl may cause lethal toxicity in half, while a higher dose, 600 mM of NaCl, was needed for Beach genotype. These lethal limit data were used to guide the salt treatment for the following experiment.

The seeds were germinated in a growth chamber (Percival Scientific Inc., Perry, IA, USA) and the healthy seedlings were transplanted into flats filled with soil. Briefly, seed coat was sliced to facilitate germination and placed on moist filter paper on a petri dish. 3-day post germination, the seedlings were transplanted in 3 × 6 flat (3.10′ × 3.10′ × 2.33′) (Greenhouse MegaStore, US) filled with potting mix soil. For salt treatment for RNA-seq assay, we use the dose that was slightly lower than the dose identified for the lethal limit mentioned above, thus the plants could be stressed enough but would not die. Thus, we used 200 Mm and 400 Mm of NaCl solution as a final concentration to stress Inland and Beach genotypes, respectively. For treatment, the plants were supplied with 50 mL NaCl solution per day. In parallel, plants supplied with the same amount of water were used as controls. Whole roots were sliced off from the treatment and control plants at 3 h, 24 h and 7d, respectively, after the concentration of NaCl was reached as designed and flash frozen in liquid nitrogen. All frozen tissues were stored at −80 °C for RNA extraction. Three biological replicates per collection were collected.

### RNA extraction, Library construction, and RNA sequencing

For each biological replicate, we used pooled root tissue from three plants for the library construction and sequencing, with each collected at 3 h, 24 h, and 7d, respectively. Briefly, the individual roots were ground and equal amounts of ground roots from the three time points were pooled to generate one biological replicate. Thus, three biological replicates per condition were generated. In total, twelve samples for both genotypes were used for total RNA extraction. Total RNA was isolated with RNeasy Plant Mini Kit (Qiagen, Valencia, CA, USA). Purified RNA was quantified using a Quant-iT™ RiboGreen™ RNA Assay Kit (Invitrogen Carlsbad, CA, USA) and its integrity was evaluated using an Agilent 2100 Bioanalyzer (Agilent Technologies, Palo Alto, CA, USA). One microgram of RNA samples with RNA integrity number (RIN) ≥ 7.0 (Table 1) from three independent biological replicates of each condition was used to generate cDNA libraries with insert sizes ranging from 300 to 350 bp using a TruSeq RNA Library Prep Kit from Illumina. Libraries were combined into a single pool and a 125 bp single-read sequencing run was conducted using a HiSeq 2500 instrument (Illumina, San Diego, CA, USA). Primary processing was performed on the raw reads to generate FASTQ files. RNA extraction, library construction and sequencing were performed in the Genomics Laboratory in the David H. Murdock Research Institute (Kannapolis, NC, USA).

### Pre-processing of sequencing data

The quality of the raw sequence generated from transcriptome sequencing was assessed with FastQC (https://www.bioinformatics.babraham.ac.uk/projects/fastqc/). Low quality (< 20) bases and adapter sequences were trimmed with Trimmomatic v 0.36^10^ with following parameters: *ILLUMINACLIP:* path/to/adaptor.fa:2:30:10 *LEADING*:3 *TRAILING*:3 *SLIDINGWINDOW*:4:15 *MINLEN*:36. After filtering, the remaining reads were called “clean reads” and were re-assessed with FastQC. All the results of FastQC were merged and visualized using MultiQC (http://multiqc.info). Clean reads were aligned to common bean (*Phaseolus vulgaris*) reference genome *Pvulgaris_218_v1.0* (https://phytozome.jgi.doe.gov) using RNA-seq aligner STAR software^11^. The general feature format (*gff3*) file (*Pvulgaris_218_v1.0.gene_exons.gff3*) corresponding to *Pvulgaris_218_v1.0* downloaded at Phytozome was used as an input for STAR. The options used for running STAR are: *--runThreadN* 16 *--genomeDir*/path/to/directory *--sjdbGTFtagExonParentTranscript* Pvulgaris_218_v1.0.gene_exons.gff3 *--readFilesIn* read.fastq.gz *--readFilesCommand* zcat *--outFileNamePrefix* Name *--outSAMtype* BAM Unsorted SortedByCoordinate. The STAR-resultant *.bam* files were used to estimate the abundance of uniquely-mapped reads using FeatureCounts^12^. Difference expression analyses was conducted using EdgeR^13^. Heat maps were made using heatmap.2 function of the gplots package^14^.

### Code availability

Codes that were used for data processing are included in the Methods and available as supplementary material (Supplementary File 1).

## Data Records

The project was deposited into the National Center for Biotechnology Information (NCBI) Sequence Read Archive (SRA) accession (Table 1 and Data Citation 1). The abundance count for all the samples was deposited at Gene Expression Omnibus (GEO) database (Data Citation 2).

## Technical Validation

### Quality control

**Figure 2:**
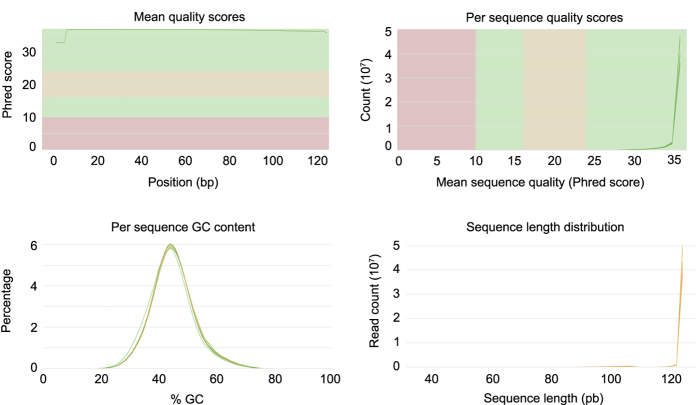
Visualization of the qualities of *S. helvola* sequencing data. (**a**) Mean quality scores per position. (**b**) Per sequence quality scores. (**c**) GC content distribution. (**d**) Read length distribution.

A total of 12 RNA libraries were prepared and sequenced with the sequencing depth ranging from 41.0–55.7 million single-end reads (Table 1). We applied FastQC to determine the data quality and measured several important parameters. The assessment for the filtered data was shown in Fig. 2, and the distribution of mean quality score and per sequence quality scores indicated the high quality of filtered sequences, with scores of most sequences over 35. Over 99% of the raw reads were kept after quality control and a 75.89–79.36% of the clean reads were mapped to unique location in the common bean reference genome *Pvulgaris_218_v1.0* (Table 1).

### Analysis of RNA-seq data

**Figure 3:**
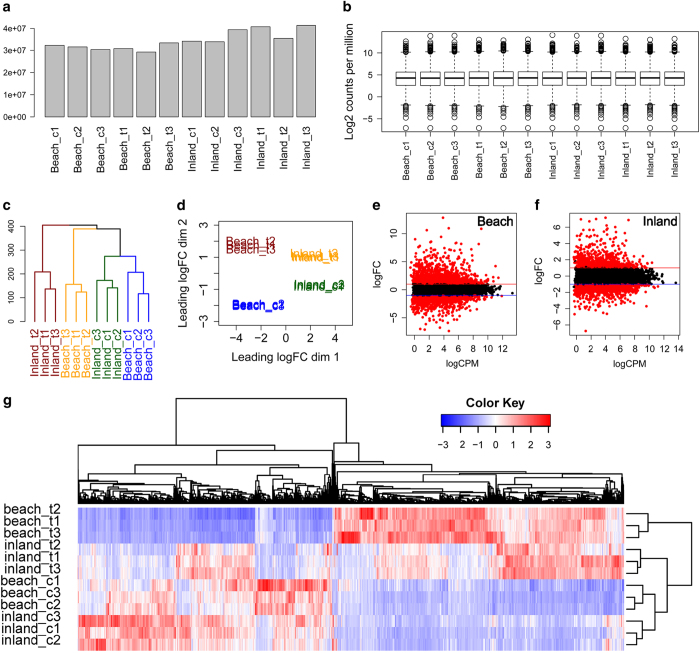
Global assessment of transcriptome data. (**a**) Library size of each replicate. (**b**) Distribution of log2 transformed count per million. (**c**) Clustering analyses of gene expression in all 12 samples. (**d**) Multidimentional scale analyses of gene expression in all 12 samples. All replicates per condition cluster together. (**e**) And (**f**) showed the MD plots of log2-expression and average abundance of each gene. Each dot represents a gene. Significantly up and down regulated genes are highlighted in red above log2(fold change)= 1 and below log2(fold change) = −1, respectively. (**g**) A heat map showing expression patterns of 2910 differential expressed genes across 12 samples.

The clean reads with single alignment on *P. vulgaris* reference exosome were counted (Fig. 3a) and normalized by counts per million (Fig. 3b) for differential expression analysis. Gene clustering analyses were used to examine the difference between the biological replicates. Figure 3c shows that three replicates from the same group cluster together while samples from different groups are well separated. This result was further supported by multi-dimensional scale (Fig. 3d) showing that gene expression profiles of all biological replicates can clearly separate the four groups and cluster biological replicates together with small variability per group. We further explored the expression profiles in two comparisons and visualized with mean-difference (MD) plots. As shown in Fig. 3e and f, a majority of the genes are cantered around the line of zero log(fold change), and the differentially expressed genes (DEGs) were highlighted in red with a threshold of fold change ≥ 2 and *fdr* ≤ 0.05. We identified 2910 DEGs in Beach genotype and have showed their expression pattern across all the samples in Fig. 3g. Consistent with Fig. 3c, replicates from each group are clustered together. DEGs showing distinct expression pattern in treated Beach genotype compared with the other three groups merit further exploration.

## Usage Notes

The RNA-seq fastq.gz files were deposited at NCBI SRA public repository and could be downloaded using fastq-dump tool of SRA Toolkit (https://www.ncbi.nlm.nih.gov). Other than Trimmomatic, FASTX (http://hannonlab.cshl.edu/fastx_toolkit/) and cutadapt^15^ are also commonly used for trimming and adapter removal. The alternative aligners for RNA-seq sequence could also be used, such as TopHat2^16^ and HISAT2^17^. The reference genome of *P. vulgaris*, the annotation file, and *gff3* file could be retrieved at Phytozome database (https://phytozome.jgi.doe.gov). For downstream differential expression analyses, Cufflinks package coupled with CummeRbund may generate transcriptome assembly, expression abundance, differential expression analyses, and visualization of analyses results. HTSeq^18^ could also be used as alternative of featureCounts for quantification and performed differential expression analyses with DESeq2^19^.

## Additional information

**How to cite this article**: Zhang, H. *et al.* Transcriptome profiling of a beach-adapted wild legume for dissecting novel mechanisms of salinity tolerance. *Sci. Data.* 5:180290 doi:10.1038/sdata.2018.290 (2018).

**Publisher’s note**: Springer Nature remains neutral with regard to jurisdictional claims in published maps and institutional affiliations.

## Supplementary Material



Supplementary File 1

## Figures and Tables

**Table 1:** Statistics analyses of transcriptomes of two *S. helvola* genotype.

Sample ID	RIN	Number of raw reads	Read length	Number of clean reads	GC %	Number of uniquely mapped reads	Accession number (BioSample)
Beach_control_1	8.4	45,285,752	125	44,993,920	43.00%	76.01%	SAMN09724906
Beach_control_2	8.0	42,584,931	125	42,352,207	44.00%	78.57%	SAMN09724907
Beach_control_3	8.7	40,982,374	125	40,734,138	44.00%	78.70%	SAMN09724908
Beach_treatment_1	7.1	42,013,548	125	41,788,983	44.00%	77.91%	SAMN09724909
Beach_treatment_2	7.4	41,437,192	125	41,226,484	44.00%	75.89%	SAMN09724910
Beach_treatment_3	7.6	45,943,168	125	45,681,909	44.00%	77.27%	SAMN09724911
Inland_control_1	7.4	47,420,589	125	47,135,101	44.00%	77.42%	SAMN09724912
Inland_control_2	8.0	45,852,012	125	45,595,609	44.00%	78.39%	SAMN09724913
Inland_control_3	8.3	53,127,911	125	52,833,664	44.00%	78.81%	SAMN09724914
Inland_treatment_1	7.9	54,434,413	125	54,103,942	44.00%	79.36%	SAMN09724915
Inland_treatment_2	7.1	47,303,936	125	47,047,987	44.00%	79.25%	SAMN09724916
Inland_treatment_3	7.9	55,728,624	125	55,425,971	44.00%	78.80%	SAMN09724917
